# Hierarchy of Loss in Functional Capability: Findings From a 15-Year Follow-Up in a Brazilian Aging Cohort

**DOI:** 10.1093/geroni/igaa057.2296

**Published:** 2020-12-16

**Authors:** Cesar de Oliveira, Dorina Cadar, Jane Biddulph, Juliana Vaz de Melo Mambrini, Fabiola Bof de Andrad, Maria Lima-Costa

**Affiliations:** 1 University College London, London, England, United Kingdom; 2 Oswaldo Cruz Foundation, Belo Horizonte, Minas Gerais, Brazil; 3 Oswaldo Cruz Foundation, Belo Horizonte, Mato Grosso, Brazil

## Abstract

Functional loss among older adults is known to follow a hierarchical sequence, but little is known about this in low-middle income countries. We examined longitudinally the hierarchy of loss in physical functioning in 1,602 older Brazilian adults (aged 60+) from the Bambui Cohort Study of Aging. Functional loss was ascertained using self-reported difficulties in six basic activities of daily living (BADL). The incidence for each BADL limitation was assessed using survival analysis while controlling for the competing risk of death. Over the 15-year follow-up, the incidence in BADL disability rate was highest for dressing, followed by getting out of bed, bathing/showering, walking across a room, using the toilet or eating. The findings from this 15-year follow-up Brazilian aging cohort support a hierarchical pattern of disability in activities of daily living based on both prevalence and incidence of disabilities over time, and they suggest gender differences in the incidence of disability.

